# 

**Published:** 1858-11

**Authors:** 


### NOTICES, &c.

WE believe that Ten Dollars a year will procure for men of culture a larger amount of current literature, characterized by scholarship, depth, comprehension, and suggestiveness, than can be obtained in any other way, by ordering of LEONARD SCOTT & Co., 79 Fulton street, New York, *Blackwood's Magazine*, *Edinburgh Review*, *London Quarterly*, *North British* and *Westminster Reviews*. To any person sending us Ten Dollars, we will send the above, and *Hall's Journal of Health* for one year.

*The Scalpel*, for October, quarterly, one dollar a year, single Nos. twenty-five cents, is, as usual, rich, racy, and rare. All that Dr. DIXON writes, medically, commands reliance, for he is a man of science. By the way, since we sent the article to the printer in which he is referred to, MULLER, the sculptor, of 437 First Avenue, has sent us a cast of *The Scalpel* man, so precisely like the original (only more so), that any of his acquaintances would recognize him in a moment. It is, for the present, on our mantel. If this skillful sculptor will send us WILLIS, and the Editor of the *Journal of Health*, in the same style, we will open a museum, and will venture a more *recherché* patronage than "BARNUM'S;" and if GREELEY and BENNETT be added, we would have to build an "extension" to accommodate the crowd—while MULLER would have his hands full of orders, immediately, if not sooner.

LETTER-CARRYING.—We believe we are doing the New York Public a real service, without fee or reward on either side, by stating that

PRICE'S LETTER-EXPRESS POST,

No. 3 Everett House, New York, on Fourth Avenue, corner of Seventeenth Street, Union Square, is the most prompt and reliable letter-carrying establishment in the city. If the person to whom a letter or package is directed, is not found, it is at once returned to the sender, if desired, by the next delivery. Up-town citizens will be glad to know, that letters are forwarded, for one cent each, to the general Post-office, four times a day, so as to meet the earliest and latest mails. To send a letter for one cent, and without fail, a distance there and back of five miles, and an hour's time saved to the sender, is certainly a great accommodation—especially, when Fahrenheit is hugging Zero, with Boreas broke loose besides.

THE REASON WHY.—We have repeatedly looked in the advertising columns of the daily papers, for one of WORCESTER'S second-hand Pianos, but to no purpose; while the "make" of this, that, and the other man, is offered at "half-price," only a

"short time" in use. We believe the reason to be, that WORCESTER's instruments are honestly made to last; and that, like a good flute, they improve in tone by age; and instead of getting ricketty from a "short time's" use, their music is sweeter—and an amateur parts with a good instrument with greater reluctance than he would with his—wife—*sometimes!*

*Godey*, for November, is noticeable for the richness of its illustrations. Dr. WILSON, of Georgia, has for several months added to the value of *Godey's* pages, by his contributions to the "Health Department." One of his instructive paragraphs closes with the following strong appeal to the women of our land: "Will not our fair readers obey these laws then, when the prize is not only beauty, with all its potent powers, but also peace of mind, ease of body, length of days, and all the countless joys, personal, social, and domestic, embraced in the word HEALTH?"

A new subscriber writes, September 21. He must be a medico-clerical gentleman, and seems to have a "vim" about which must "tell" in the course of a life-time:

"Please send me your '*Journal of Health.*' I don't care a fig for your 'health items' (for I have a distressing amount of health); but I like the ring of your articles—they leap from the die twenty-two carats, and full weight. I never read your Journal till to-day. Professor D. A. HOLBROOK, with whom I am stopping, called my attention to it. I received a shock on the first page, and now wish to strike hands with a man who has sloughed cant, and stands unlaced before his fellows. The sharp ringing dialect of common sense, which characterizes your Journal, pulls the dollar from my pocket, and a strong '*God bless you!*' from my heart. Take the dollar, and send the Journal. By it I hope to keep the adversary ('Throat-Ail') from my pulpit, and ennui from my people."

SEWING-MACHINES.—The liberality of WHEELER & WILSON, in furnishing clergymen's wives their superior Sewing-Machines at half-price, is worthy of commendation. The public will be gratified still further to learn, that the company has got out a new pattern, for FIFTY dollars. It will be remembered, that WHEELER & WILSON's Machine makes the smoothest and strongest stitch of all its competitors.

One of the most appropriate weekly publications we know, for families, where culture, grace, and refinement preside, is the *Musical World*, edited by R. STORRS WILLIS, New York. $2 a year.

We are indebted, for the *Minutes of the Medical Society of North Carolina*, to the courtesy of that excellent physician, S. S. SATCHWELL, M. D., of Newberne, N. C. The Society numbers 172 members.

